# Gaming in the intervention and support process: A realist evaluation of a gaming-based programme

**DOI:** 10.1177/13623613251320542

**Published:** 2025-03-15

**Authors:** Melissa H Black, Abi Lilford, Vy Nguyen, Erin Walker, Haw Huei Wee, Olov Falkmer, Sarah McGarry

**Affiliations:** 1 Curtin University, Australia; 2 Karolinska Institutet and Region Stockholm, Sweden; 3 Humdrum, Australia

**Keywords:** autism, social connection, strengths-based, video-games

## Abstract

Using games as part of the intervention and support process (sometimes referred to as ‘gaming therapy’) is an increasingly popular method of supporting autistic youth to develop social competencies and other skills because they are believed to be inherently motivating and fun for youth. Some emerging research has indicated that intervention programmes using games can be beneficial for autistic youth, but little is currently known about why, how and for whom they work. This study conducted a realist evaluation of a programme using the video game “Minecraft” for autistic youth, comprising interviews and observations with autistic youth (*n* = 4), facilitators (*n* = 2) and caregivers (*n* = 6). Findings from interviews and observations suggest that leveraging strengths, sharing power by placing youth in the ‘expert role’ and learning in a naturalistic and motivating environment provided youth with the opportunities to develop social competencies in communicating their wants and needs and supported the development of social connections, independence and emotion regulation. The results of this realist evaluation may provide a framework for future intervention and support programmes using video games.

**Lay abstract:**

Using games as part of the intervention and support process (sometimes called ‘gaming therapy’) is increasingly used with autistic youth. Gaming is believed to be fun, motivating, and helpful in supporting autistic youth to develop social connections and skills, but very little research has explored this. In this study, we looked at a gaming-based programme that uses Minecraft. We did interviews and observations with youth, facilitators and caregivers of youth to find out why, how and for whom it works. We found that the gaming-based programme could be particularly motivating for youth interested in video games. Facilitators shared the interests of the youth and shared power by placing youth in the ‘expert role’, while providing youth with opportunities to learn and develop in their own way. Feedback from youth, their caregivers and facilitators suggests that the gaming-based programme might help youth develop social connections, independence and emotion regulation. The results of this realist evaluation may provide a framework for future gaming-based programmes.

Autistic youth can face many social challenges that can have negative and long-term consequences on mental health (Ratcliffe et al., 2015; Schiltz et al., 2021), employment (Harmuth et al., 2018) and overall community participation (Haertl et al., 2013). While autistic individuals commonly report a desire for friendships, they frequently indicate having few or no friends, lower quality friendships and more feelings of loneliness than neurotypical counterparts (Umagami et al., 2022). Autistic individuals can also experience anxieties about their social performance (Black et al., 2023) and are more likely to have clinically diagnosed social anxiety disorder (Spain et al., 2018).

While research and practice has previously suggested that social interaction challenges experienced by autistic individuals were solely due to ‘deficits’ within an individual, more recent framings, particularly the neurodiversity paradigm, propose that autistic individuals are not ‘deficient’ in social skills but rather have experiences that differ from the majority (non-autistic) (Dwyer, 2022). Contemporary research now points to both individual (e.g. monotropic tendencies, differences in social cognition) and contextual (e.g. the mismatch between autistic and non-autistic communication styles) factors that contribute to the social challenges experienced by autistic individuals (Crompton et al., 2020; Isaksson et al., 2019; Murray et al., 2005). While improving community acceptance and awareness of autistic communication styles seems essential for addressing broader social challenges experienced by autistic individuals (Levi et al., 2023), supporting the development of social connection, social competencies and social confidence is also required, particularly due to demands to navigate a primarily non-autistic world.

Autistic individuals report difficulties in areas such as expressing themselves, understanding verbal and non-verbal information, misunderstanding (neurotypical) social rules and difficulties initiating and/or maintaining social interactions (Gurbuz et al., 2019; Levi et al., 2023; Müller et al., 2008). Indeed, support with ‘social skills’ was found to be a priority for future research among autistic adults (when ranked on a 5-point Likert-type scale, with 5 being very important social skills training received a rating of 4.16+) (Gotham et al., 2015), with a recent systematic review finding that social skills interventions, among other factors, were associated with decreased loneliness in autistic individuals (Umagami et al., 2022).

Traditional social skills interventions are commonly employed to support autistic youth and adults to manage social obstacles (Hull et al., 2024; Radley et al., 2020). These social skills training programmes typically involve groups of autistic individuals learning and practicing social skills under the guidance of therapists acting as facilitators. These programmes have shown efficacy in promoting social competencies (Radley et al., 2020). However, they also have limitations. These interventions show limited generalization of skills to other contexts (Moody & Laugeson, 2020), and their formats may not be engaging for autistic youth, or may not fully meet their needs. Indeed, autistic adults have reported that while support with social skills was wanted, currently available programmes may not address their needs, expressing a desire for social skills support that is interactive, relevant, personalized, inclusive and holistic (Hull et al., 2024). Further, some targets or teaching strategies in more traditional interventions, though well-intentioned, may inadvertently act to promote camouflaging by promoting the learning of specific neurotypical behaviours (for example by encouraging eye contact) (Mandy, 2019) or may erode self-esteem by focusing exclusively on deficits (Pellicano & den Houting, 2022). More contemporary intervention frameworks now seek to move towards approaches that are more accepting of neurodiversity and look beyond ‘deficits’ to instead more holistically recognize and leverage strengths (Pellicano & den Houting, 2022).

The implementation of video games in the intervention and support process, sometimes referred to as ‘gaming therapy’ has been proposed as an alternative to more traditional intervention formats to support social connection and skill development for autistic youth. Games can tap into areas of interest and strength for youth and are proposed to be more inherently motivating and fun, potentially increasing engagement in the intervention process (Clough & Casey, 2011; Silva et al., 2021). In addition, games using multiplayer functions offer a wide range of opportunities for individuals to enhance their experiences of social connections in more naturalistic contexts, replicating what youth do in their everyday lives (Finke et al., 2018). The use of games in the intervention and support process can span from the use of commercially available entertainment games to ‘serious games’ that target particular skills and are specifically designed for clinical practice (Ahmed et al., 2023; Silva et al., 2021; Tang et al., 2019). The majority of research to date has focused on serious games, but both serious games and commercially available entertainment games (for example, Minecraft and Wii Sports) have shown efficacy in supporting autistic youth to develop a range of skills (Silva et al., 2021). Specifically, a recent review of the literature demonstrated that game-based interventions show efficacy in promoting social skills among autistic children and youth (Walsh et al., 2024). Although the use of games within the intervention and support process can still focus on ‘symptom reduction’(Silva et al., 2021), their naturalistic and strengths-based approach also has the potential to support more neurodiversity-affirmative social skills interventions.

Here, we describe a gaming-based programme that uses ‘Minecraft’ (https://www.minecraft.net/en-us), a free-roaming game that can be played cooperatively or individually. Minecraft is a sandbox construction game that includes survival elements, where individuals are free to create and explore (See Duncan & Sean, 2019 for a more detailed explanation). The popularity of Minecraft among youth, combined with its sandbox design and multiplayer capabilities, makes it an attractive programme for intervention and support applications where therapists may be better able to build rapport, harness a youth’s strengths and interests, and more implicitly develop skills and connections in a naturalistic context. Minecraft has received some attention in neurotypical youth contexts, where it has been applied in educational settings, where the game-based learning has been suggested to facilitate communication and skill development (Hewett et al., 2020; Niemeyer & Gerber, 2015). Minecraft has now also become more popular for supporting autistic youth, with therapists and service providers beginning to incorporate variations of Minecraft into their services, with some sharing anecdotal evidence pointing to its effectiveness (Finch, 2021; Montali, 2022; Zolyomi & Schmalz, 2017). However, there is limited scientific investigation of the use of Minecraft and entertainment games more broadly in intervention and support contexts. One study examined the use of Minecraft to support social skill development of four autistic boys (aged 11–13 years) participating in a study at a university campus. Based on video observations, some evidence was found to support that autistic youth made more social initiations in later sessions of the intervention (MacCormack & Freeman, 2019). Another case study examined how Minecraft could be used in the treatment of an 11-year-old autistic boy who experienced trauma, finding some evidence that it may be helpful (Gerhardt & Smith, 2020). Given that entertainment games, especially Minecraft, are being incorporated into service provision but have received limited empirical investigation, greater exploration and understanding of these interventions are required, especially if they are to be implemented in a strengths-based and neurodiversity-affirmative way. Here we examine a gaming-based programme for autistic youth using a realist evaluation method. This method enables an in-depth exploration of the outcomes of the gaming-based programme, as well as the mechanisms (what works) and contexts (conditions and circumstances) that drive outcomes.

## Methods

### Research design

A qualitative, ethnographic approach comprising interviews and observations, guided by a realist evaluation was employed to determine the contexts, mechanisms and outcomes (CMOs) of a gaming-based programme delivered by a therapy service provider (Pawson & Tilley, 1997). Gaming-based programmes are complex interventions, which are defined as interventions having multiple components and complicated causal pathways that interact to produce outcomes (Skivington et al., 2021). Realist evaluations provide a method for evaluating complex interventions focused on identifying and describing the interplay between the contexts, mechanisms and outcomes of an intervention, acknowledging that the outcomes of an intervention can be influenced by specific factors within an intervention, as well as broader contexts of an individual and their environment (Pawson & Tilley, 1997).

### Service provider and gaming-based programme

Humdrum is an Australian National Disability Insurance Scheme (NDIS) registered organization located in Perth, Western Australia that provides a variety of services such as occupational therapy, speech pathology, physiotherapy, behaviour support and other services for neurodivergent individuals. The gaming-based programme is one service offered by Humdrum to support neurodivergent youth, particularly autistic youth, but also other youth with social needs, to develop their social competencies (https://humdrum.community/).

The gaming-based programme sessions at this service are typically conducted once a week for 1 hour online and are conducted in groups of up to three youth and facilitated by a therapist (for example occupational therapists or speech pathologists). The gaming-based programme at Humdrum is a structured framework and model of intervention and support using Minecraft to facilitate social competency development. In this framework, four roles are created (miner, crafter, builder, guard) and each player takes on one role. A miner is able to gather resources from the environment, such as wood or stone. The crafter is able to use the gathered materials to craft in-game items, and the builder is able to use the gathered materials to build structures. The guard is able to fight monsters to protect other plays while gathering and crafting. As players can no longer access all functions of the game, they are required to communicate and work as a team to achieve goals. Facilitators work with youth to develop goals that the groups will work towards (for example building a house). Facilitators then play alongside the youth, allowing youth to engage and interact to achieve their goal, and providing input to assist in developing skills as difficulties emerge. For example, working on communicating frustration or needs to group members, or encouraging emotion regulation strategies. In some groups, ‘leader’ and ‘follower’ roles are used whereby, for a particular session, a participant undertakes a leadership role that encompasses task delegation and decision-making, while the follower role requires the use of listening and probing skills to assist the leader in achieving session goals. The facilitator process employed is highly personalized, depending on the specific group composition and individual needs. As a first line, therapists used strategies such as modelling, coaching or facilitating problem-solving through probing to encourage youth to problem-solve and develop solutions to issues arising (for example by asking questions such as ‘how could you express what you need to your peer?’, ‘what is a way that you could let your peer know that you are frustrated?’, ‘how could we come up with a solution together?’). More explicit instruction was, however, used when required depending on the specific need, for instance, in cases of significant disagreement. In cases of significant disagreement or conflict, facilitators had the ability to virtually ‘teleport’ all players to one location in the game for discussions, the virtual equivalent of changing environments within face-to-face delivery.

### Participants

Participants included autistic youth attending the gaming-based programme through the service provider, their caregivers and facilitators of the programme. To be eligible for inclusion, autistic youth were required to be participating in the gaming-based programme at the service provider. Participants with a range of clinical diagnoses were eligible for inclusion, such as autism or attention deficit hyperactivity disorder (ADHD). However, only youth with a primary diagnosis of autism opted to participate in the study. Caregiver participants were required to be guardians or caregivers of youth attending the gaming-based programme, and facilitators were required to be providing the gaming-based programme at the service.

A total of 12 individuals participated in this study including four youth, six caregivers and two facilitators. The six caregivers discussed six children, but two children declined to participate in the study and did not engage in interviews or observations. Thus parent-report data is only available for these children (a 10-year-old autistic female with co-occurring ADHD, social anxiety and general separation anxiety and a 6-year-old autistic male). Realist evaluations emphasize flexibility in sample sizes depending on the specific contexts and complexities of the subject of evaluation (Wong et al., 2016). For this reason, the sample size was deemed appropriate to achieve sufficient depth and breadth in identifying the context, mechanisms and outcomes of the gaming-based programme. Thematic saturation was achieved.

Autistic youth participants who engaged in interviews and observations were all male and were aged between nine and 17 years of age. One autistic youth had a co-occurring speech and language delay. All youth were currently receiving other therapy services including occupational therapy (*n* = 4), speech therapy (*n* = 3) and physiotherapy (*n* = 1). Caregivers were primarily females and mothers (*n* = 5) and one male father (*n* = 1). Caregiver ages ranged between 39 and 54 years. All caregivers reported being married and living with a partner and child/ren. Four caregivers were employed full-time and two were employed part-time. All caregivers had at least a high school education, two had postgraduate degrees. Therapists were one male and one female. One held an undergraduate degree, and one held a postgraduate degree.

### Materials

#### Sociodemographic questionnaire

A sociodemographic questionnaire was used to collect basic demographic characteristics of participants. The sociodemographic questionnaire included questions pertaining to participants’ age, gender, diagnoses and education levels. This information allowed us to characterize the sample.

#### Interview and observation guide

A semi-structured interview guide was developed for the purpose of this study. Questions were created specifically for each participant group regarding their experiences with the gaming-based programme, centred on examining the contexts, mechanisms and outcomes of the programme. A copy of the interview guide can be found in the supplementary material. An observation guide was used during each observation session. This allowed the observer to take notes in a structured manner under the relevant subheadings; physical setting, activities, social environment, informal interactions or unplanned activities, non-verbal communication and observing occurrences with the sessions. It also served as a reminder of key areas of interest which are the CMOs of the gaming-based programme.

### Data collection

All participating autistic youth and facilitators were provided the option to be interviewed, observed, or both, while caregivers were interviewed. Semi-structured interviews were completed online through video calls or phone calls. The interviews explored their views, opinions and perceptions of the gaming-based programme. Interviews were audio-recorded except for one caregiver interview where consent to audio-recording was not provided. In this instance, written notes were taken by the researcher. Observations of the four autistic youth and two facilitators were undertaken over seventeen observation sessions by one to two researchers. During the observations, researchers observed the interactions between the consenting autistic youth and facilitators, with observations according to the observation guide journaled.

### Data analysis

Interviews were transcribed verbatim, and observations and interview transcripts were imported into NVivo for thematic coding (QSR International, 2018). Braun and Clarke’s six stages of thematic analysis were employed: (a) data familiarization, (b) initial code generation, (c) producing themes, (d) reviewing themes, (e) labelling themes and (f) report writing (Braun & Clarke, 2006). Data were analysed iteratively using both deductive and inductive approaches. First a deductive approach was undertaken where data were broadly coded into three categories according to the Realist Evaluation Framework: context, mechanism and outcome (Pawson & Tilley, 1997). Within this grouping, data were allocated to a context category if it referred to aspects present before commencement of the programme, including characteristics of the person and the environment. Data were allocated to a mechanism if it referred to elements within the programme, while data were allocated to an outcome category if it referred to predicted and unpredicted consequences of the programme. Example quotes for each context, mechanisms and outcome category are provided in the supplement. An inductive and iterative coding approach was then utilized whereby quotes and observations were examined to generate codes, and subsequently themes, within each of the three groupings. Themes were further analysed and coded using a combination of deductive and inductive approaches to demonstrate the relationships arising between contexts, mechanisms and outcomes. Throughout the analysis, both representative and discrepant data were examined, focusing on exploring how they influence the context-mechanism-outcome relationships.

Several methods were used to enhance trustworthiness. First, credibility was ensured through triangulation of findings across the three participant groups, and the combination of interview and observation data collection methods. Researchers also immersed themselves in the programme, undertaking an extended observation period (Lincoln & Guba, 1985). Transferability was ensured by providing a rich description of the service, the gaming-based programme and the findings (Lincoln & Guba, 1985). Dependability was ensured by keeping an audit trail. The audit trial and triangulation also supported confirmability. Research bias was also addressed through the application of the semi-structured and structured observation guides which ensured consistency in data collection. Researchers also regularly engaged in peer-debriefing throughout data collection and analysis to discuss insights as they developed and to engage reflexively with assumptions and biases.

### Ethical considerations

Human Research Ethics Committee approval by Curtin University was obtained (HRE2021-0700). An information sheet regarding the study was provided to participants and their caregivers and written assent and consent was collected from the child and guardian respectively. To ensure confidentiality, all participants’ names were replaced with pseudonyms.

## Findings

**Figure 1.**
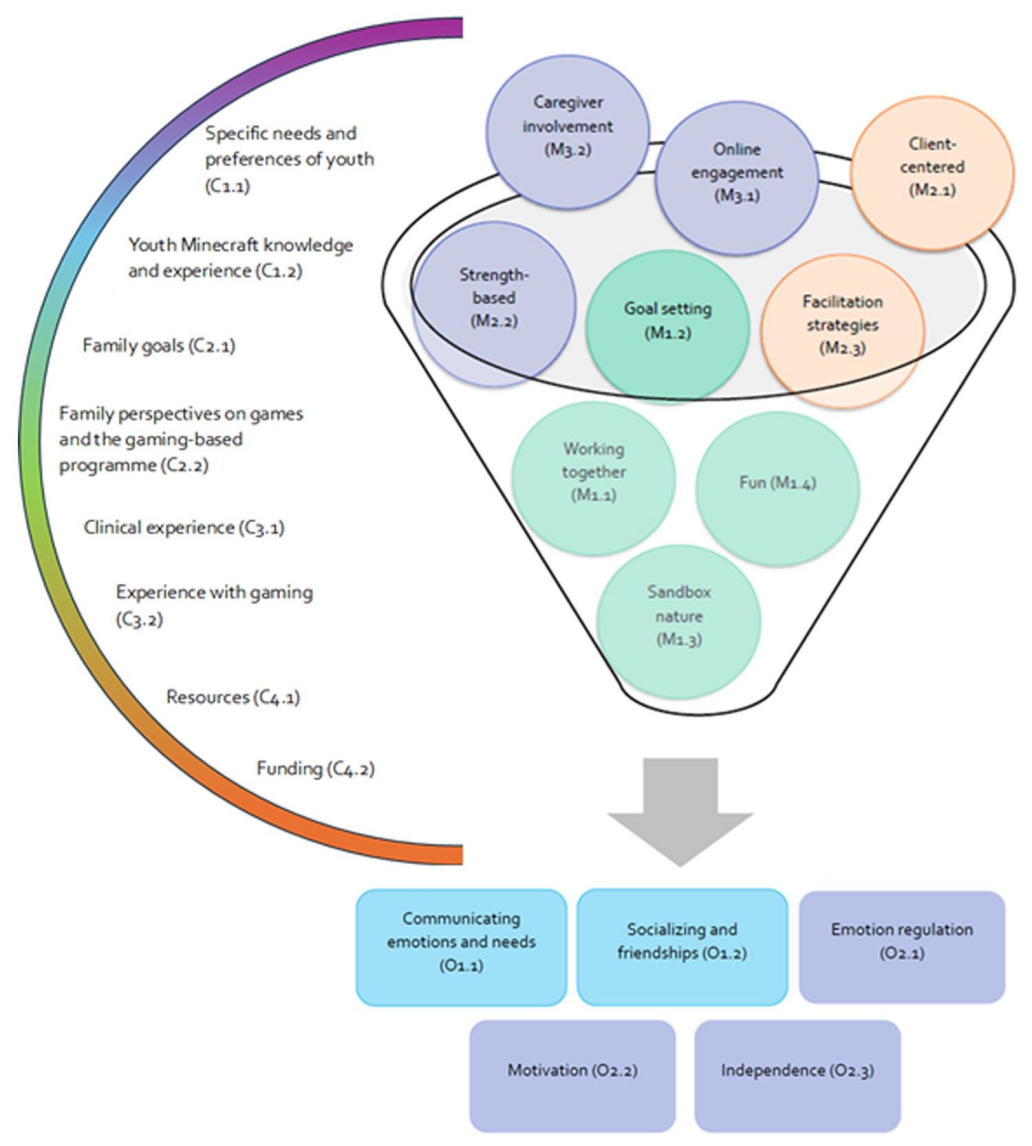
Overview of realist evaluation findings.

The analysis found eight context codes, nine mechanism codes and five outcome codes which were arranged according to four context, three mechanism and two outcome themes (Figure 1).

### Contexts

#### Autistic youth background (C1)

This theme represented aspects and characteristics of the youth before the programme, including specific needs and preferences (C1.1), and Minecraft knowledge and experience (C1.2). The youth joined the gaming-based programme with a range of different needs, requirements of the programme and preferences (C1.1). Youth had various strengths, challenges and differences, but common difficulties relevant to the programme included emotional management skills and social communication. Some youth experienced difficulties expressing emotions during emotional distress or conflicts, difficulties understanding the perspectives of their peers, and difficulties sharing ideas and providing instructions to others. Some youth had different preferences and needs, for example, one caregiver highlighted that their child often preferred participating at home where it was quieter and easier for the participant to engage. Another caregiver stated that leaving the house to attend services could negatively impact their son. Youth in the gaming-based programme often had previous experience with computer games and had played Minecraft previously, having existing skills, abilities, and interests in this area (C1.2). Some youth were reported to be more knowledgeable about Minecraft and gaming than the facilitators.

#### Caregiver background (C2)

This theme represented the background of caregivers and included their family goals (C2.1) and perspective on games and the gaming-based programme (C2.2). Family goals (C2.1) entail the caregivers’ desired outcomes for their children following participation in the gaming-based programme. Caregivers expressed that they would like the programme to help their child to develop social and communication skills. Examples include ‘asking for help, taking turns, giving instructions or giving directions he really struggles with that’ and ‘getting the kids to take on roles and make decisions and that kind of stuff which the boys struggle with’. One caregiver reported that they hoped for their son to learn how to communicate his emotions through the programme so that he is ‘communicating correctly and telling me the honest truth . . . be able to communicate and not get aggressive’. Other goals also include independence skills. Two caregivers had expressed that they would like their child to be less reliant on them and be more independent.

Caregivers had mixed feelings regarding the use of games in the intervention and support process (C2.2). Most caregivers could see the value of using games, believing that they could provide their children with the opportunity to develop their social skills and confidence. One caregiver described that ‘80% of gaming therapy is teamwork, listening and understanding while the 20% is the game itself’. However, some caregivers also expressed concerns that their children already spent too much time gaming and also expressed concerns about online safety.

#### Facilitator background (C3)

This theme represented the aspects of the facilitators and included their clinical experience (C3.1) and experience with games (C3.2). The facilitators had various clinical experiences working with neurodivergent individuals (C3.1) that they used to inform their support and intervention approaches. Facilitators spoke about how their knowledge of neurodivergent conditions influenced how they tailored the instructions provided in the game and the extent to which they challenged youth in the programme. Both facilitators spoke about drawing from other relevant clinical experiences, such as using Lego-based therapy and floor-time therapy. Experience with games (C3.2) refers to facilitators’ past experiences and knowledge of gaming and Minecraft. One therapist reported that he had knowledge of the game prior to beginning the gaming-based programme. He explained that he had played this game with his friends from home and found it to be an ‘amazing’ tool before introducing it. In contrast, the other facilitator was unfamiliar with Minecraft and explained that they had to learn quickly how the game works due to COVID and telehealth. Each facilitator was required to build the skills to provide services online which has increased the success of engagement and interaction with the participants.

#### Resources (C4)

This theme represented the institutional factors and included: Resources (C4.1) and funding (C4.2). To participate in this programme, participants were required to have the resources and access to technology to connect with therapists and other participants online to play the game as well as the appropriate environment for the sessions to take place (C4.1). Participants required a laptop or computer with an Internet connection, a separate device for video calling and an area where they could attend the programme. Therapists also required these resources to facilitate sessions. Funding refers to the funding that participants can spend on disability services and resources provided by funding plans (C4.2). Caregiver interviews revealed that the availability of funding for engaging in the gaming-based programme was one of the main factors influencing their child’s participation. One caregiver explained that having programme sessions online was helpful because it removed therapist travel costs leaving funding for other interventions.

### Mechanisms (M)

#### Activity design (M1)

This theme represented the mechanisms relating to the design of the activities within the programme sessions. This theme contained five mechanisms; working together (M1.1), goal setting (M1.2), sandbox nature (M1.3) and fun (M1.4). Within sessions, facilitators and youth were required to work together to achieve goals (M1.1). One youth participant shared that the gaming-based programme was ‘quite a bit more like socially dependent than in like, other just casual gameplay’. To facilitate working together, roles within the game (miner, crafter, builder and guard) were allocated to the participants. To actively participate within their allocated roles, participants were required to communicate with each other and work as a team to complete objectives. One youth participant noted that they ‘had to work together to get what we need to do’. In some cases, depending on the needs of specific groups a ‘leader’ was selected who would be responsible for the allocation of work and provide instructions to the rest of the team.

In-game goal setting (M1.2) was completed at the start of each session. Goal setting was completed in two ways depending on the needs of the specific groups. First, facilitators worked together with participants to enable them to collaboratively develop a shared goal. Alternatively, a leader specified a goal for the whole team. Once a goal was created, smaller objectives were discussed within the group, either spontaneously or facilitated by the therapist and continuously discussed while playing. Goals are flexible and open; short goals may result in multiple goal setting within the session and longer goals may take a few sessions.

The sandbox nature of Minecraft (M1.3) was described by a facilitator as the ability to ‘. . . decide exactly what it is that want to do and how you want to do it’ which meant that participants could decide for themselves ‘the resources needed, timeframes and priorities’. In this way, youth were able to develop a range of different goals for sessions and could develop their own plans for how to achieve their goals. One facilitator described that the sandbox nature of Minecraft provided ‘more accurate representation of actual play or play that is away from the screen’ where youth were required to navigate the Minecraft environment where ‘spoken and non-spoken social rules exist’.

The programme sessions were described as fun by the youth (M1.4). One youth participant described the gaming-based programme as a ‘fun spin on the usual experience’. Caregivers also observed that facilitators ‘didn’t push them [the children] to [attend the programme sessions], or bribe, motivate them. They’re doing it themselves’, while another caregiver reported ‘[my son] loved everything about it. It’s the only therapy [he] doesn’t moan about’. This was echoed by a facilitator who shared ‘it means that we don’t have to start with rewards and then taper back. Instead, we’re doing something that is truly, truly rewarding’ Facilitators shared that they believed that the fun nature of the gaming-based programme impacted their service provision whereby youth felt comfortable and more confident in their abilities, enabling facilitators to challenge the participants..

#### Facilitator approach (M2)

This theme represented the mechanisms relating to the facilitators’ approaches to working with youth in the gaming-based programme: Client-centred (M2.1), strength-based (M2.2) and facilitation strategies (M2.3). Client-centred approach (M3.1) referred to the facilitators adapting their approaches to the specific needs of the participants for optimal engagement and learning. One facilitator spoke about the importance of being flexible with their clients ‘to work through problems, not create more problems for them’. They emphasized the importance of knowing how to adapt and compensate for certain skills while working on others so to create the just-right challenge. The strengths-based approach (M3.2) refers to therapists’ ability to support and complement the autistic youths’ existing capacities and strengths. Participants’ existing Minecraft skill sets were leveraged by therapists to create an engaging programme. A facilitator noted that it was beneficial when youth had knowledge in Minecraft because it created opportunities for them to teach and give instructions to the facilitators. They also described that ‘Minecraft is a familiar environment where the participants have competencies and skill sets’ allowing participants to feel more confident as they do not view the programme as being overly challenging as they ‘effectively compensate with their other skills’. Facilitation of activities (M3.3) refers to the therapists’ skills in facilitating skill-building activities which may involve prompting, guiding and modelling. As observed throughout sessions, facilitators would often model communication and play skills first, demonstrating how it may look like to the participants. The facilitators would then prompt, question and guide the participants when necessary to encourage them to practice and enhance their learning opportunities. Positive reinforcement was observed through acknowledgement and praise from facilitators. Facilitators also demonstrated their expertise in finding learning opportunities throughout the session, posing appropriate questions to facilitate reflection and internalization of knowledge.

#### Environment (M4)

This theme included online engagement (M4.1) and caregiver involvement (M4.2). Online engagement (M4.1) referred to Minecraft’s capacity to create online servers for online gaming-based programme sessions. Caregivers described that the online groups could benefit their child and found that having the programme online at home has been more convenient for their time management and attendance. Furthermore, online sessions removed the constraint of location, allowing the gaming-based programme to reach more remote regions. However, the online nature of gaming-based programme was noted by one facilitator to not be appropriate for all youth, one facilitator shared: ‘if your client is someone who needs to have like face-to-face co-regulation, then it might not be a great idea’.

The online nature of the programme meant that caregivers could become involved in the programme (M4.2). One facilitator shared that this could be beneficial if youth require assistance with co-regulating emotions: ‘it’s always good to have an adult floating around . . . especial when some of our clients have difficulties with regulating their emotions’. While caregiver involvement also allowed caregivers to learn strategies to that could benefit their child at home. While some level of parental engagement could be beneficial, too much engagement from parents can have the opposite effect whereby ‘some parents take over a bit too much’ and intervened to solve conflicts or disagreements which could have been learning opportunities for the youth. Some caregivers chose not to be involved because their child become reliant on them for help ‘If I’m around or my husband’s around, [child’s name] will rely on us to do stuff for him’.

### Outcomes (O)

#### Social competencies and connections (O1)

The generated outcome codes of this theme are about skills the adolescents developed from participating in the gaming-based programme: Communicating emotions and needs (O1.1), and socializing and friendships (O1.2). Communicating emotions and needs (O1.1) referred to youth’s ability to express their needs and emotional states with others. One caregiver reported that they believed that their child was better able to communicate his feelings after participating in the gaming-based programme, for example ‘[he] will tell his friends . . . you’re really upsetting me’. Youth were also more able to express their needs in social settings. One caregiver explained that ‘[her son] speaks more loudly and clearly when he’s gaming, and that has transferred over into when he’s gaming with other kids as well online’ and with other people in real life he ‘speaks a lot louder and more clearly so you can understand what he’s saying’.

Socializing and friendships (O1.2) referred to the youth’s ability to communicate, take turns and participate in play with their peers or facilitators during and outside of sessions. One youth participant noted that they were more able to ‘socialise and talk with each other’ and ‘coordinate with each other’ in-game to achieve goals. Another youth participant noted that his ‘general communication’ and ability to talk to people had improved since engaging in the gaming-based programme. Some youth participants started playing video games with others, with one caregiver reporting that their child had started to ‘create teams rather than playing solo all the time . . .’. when playing video games. Caregivers, facilitators and youth also shared that some youth had developed friendships within the programme sessions that had extended outside of the sessions once they had concluded.

#### Other benefits (O2)

Emotion regulation (O2.1), motivation (O2.2) and independence skills (O2.3) were other described benefits of the gaming-based programme. Youth were reported to demonstrate improvements in their ability to regulate emotions and employ emotion regulation strategies. For example, one caregiver reported that their son would say ‘I’m going to go’ instead of throwing his ipad across the room’. Motivation (O3.2) referred to the participants being intrinsically motivated to engage in the programme. The youth were incentivised to actively engage in the programme by having the opportunity to play a ‘fun’ game that aligned with their interests, rather than completing ‘work’. One facilitator described that their participants began to perceive engaging in the service as fun and enjoyable rather than under stimulating or tedious. This view was shared by caregivers who noted that their children enjoyed the gaming-based programme because the game was fun and something they were already interested in. Independence refers to the child’s capacity to demonstrate initiative (O3.1). One caregiver reported an improvement in their child’s autonomy with sessions as they were ‘taking the initiative to do those things, which was not normally what they would do, taking charge’ and ‘taking on roles and making decisions . . .’.

### Context-mechanisms-outcome combinations

The contexts and mechanisms identified in this study showed interactions contributing to the observed outcomes. The interests of the youth (C1.2) were leveraged within the programme by using Minecraft, which was creative (M1.3) and fun (M1.4). By leveraging the sandbox and fun nature of Minecraft, facilitators employed strengths-based approaches (M2.2) that harnessed the strengths and interests of the youth. These approaches led to youth feeling more engaged and motivated in the intervention process (O2.2). At the same time, because a strengths-based approach (M2.2) based on the interests of the youth (C1.2) was employed, the facilitators were able to challenge the skills of the children (M3.3), enhancing outcomes (O1, O2). In supporting the youth, facilitators were required to use their clinical experience (C3.1) and client-centred approaches (M2.1) to adapt their facilitation strategies (M3.3) to the social competencies (C1.1) and specific needs and preferences (C1.2) of the autistic youth, as well as the goals of the families (C2.1). Being involved in goal setting (M1.2), working together (M1.2) and strengths-based approaches (M2.2) that placed youth in an ‘expert role’ because of their previous gaming experiences (C1.2) provided youth with opportunities to develop their independence skills (O2.3). This was particularly the case where facilitators were less familiar with Minecraft (C3.2). Working together to achieve goals (M1.2), combined with facilitation from the facilitators (M3.3), also provided youth with opportunities to socialize and develop skills in communicating their emotions and needs (O1.1), and socializing and forming friendships (O1.2) and emotion regulation (O2.1).

The online nature of the gaming-based programme (M4.1) supported some of the youths’ specific needs and preferences (C1.2). Especially if they preferred being at home or online, contributing to motivation to engage (O2.1). Because youth were at home, caregivers could become involved in the programme sessions (M4.2), but this was influenced by the caregiver’s perceptions of games (C2.2), and too much caregiver involvement could impact independence (O2.3) and emotion regulation (O2.1) skill development.

## Discussion

This study examines the active ingredients contributing to outcomes of a gaming-based programme using Minecraft for autistic youth using a realist evaluation approach (Pawson & Tilley, 1997). By understanding the context-mechanism-outcome framework, this study identified why and how a gaming-based programme using Minecraft works within a service provision context and who this intervention format might be beneficial for. Identifying and understanding these elements can help inform the development and design of future gaming-based programmes.

The gaming-based programme utilizing Minecraft leveraged the strengths and interests of the autistic youth, and all youth participating in the study had previous experience with Minecraft and gaming. The youths’ interest in gaming was a key underlying factor influencing their continued engagement in the intervention process, and facilitators leveraged the experiences that youth had with Minecraft, placing them in the ‘expert role’. Placing youth in this expert role differs from other more traditional interventions or similar skill development programmes, which tends to emphasize difficulties. Although the overall aim of the current gaming-based programme was similarly to support youth to develop areas of challenge, it did so by also recognizing and harnessing the youths’ experiences and knowledge. The gaming-based programme employed may therefore be considered ‘strengths-based’. Strengths-based practices seek to enhance an individual’s existing strengths or use strengths to overcome or develop areas of difficulty (Urbanowicz et al., 2019). Results from the current study suggest that employing such a strengths-based approach may have been beneficial in providing an environment where youth feel confident to practice and develop skills. Indeed, facilitators in the current study reported that they felt they were able to present more challenges to the autistic youth to support the development of skills. Youth were also motivated and engaged to participate in the programme.

Related to the strengths-based nature of the programme, an important component of the gaming-based programme was that facilitators supported power-sharing with youth during the programme, providing options for decision-making. This shared power further assisted in placing autistic youth in the ‘expert role’ and encouraged youth to use their own initiatives to develop goals and problem-solve solutions. This increased requirement for youth decision-making and initiative seemed to be necessary for promoting independence Resultantly, though having some previous experience with gaming and Minecraft may influence how well facilitators may be able to immediately engage with and share with youth and their interests to develop rapport, being able to share power and place youth in the ‘expert role’ may be more important to supporting overall outcomes.

Unlike more traditional social skills group training programmes, the gaming-based programme utilizing Minecraft also took a more implicit approach to assisting youth in developing their social competencies and social connections. Some more traditional social skills training programmes can be embedded in behaviourist models that seek to encourage autistic individuals to demonstrate neurotypical social behaviours (Mottron, 2017). Although these programmes do show effectiveness, some approaches have also attracted criticism over recent years because of the potential for these interventions to encourage camouflaging, which can be associated with negative mental health outcomes (Mandy, 2019). The social competencies that facilitators supported youth to develop in the evaluated programme were instead not related to neurotypical standards, but rather related to providing strategies and supports to communicate ones’ emotions and needs in their own way. As such, the gaming-based programme employed may represent a more neurodiversity-affirmative approach to assisting autistic youth in developing social competencies because they do not seek to encourage ‘non-autistic’ behaviour or communication but instead, provide strategies and supports to enable an individual to develop autonomy, communicate their needs and wants in their own ways and socially connect with others how and when they like.

The gaming-based programme was reported by caregivers, youth and facilitators to assist in the development of several social and non-social competencies, such as enhanced abilities to communicate emotions and needs, forming and maintaining social connections, regulating emotions and independence skills. Importantly, however, there are several contextual and mechanistic factors that may act to influence whether the outcomes reported in the current study are observed. For example, the gaming-based programme leveraged the strengths and interests of the youth in games and Minecraft, and this was a key factor motivating youth and providing a framework of strength that facilitators could leverage when working with youth to develop competencies in areas where they may experience challenge and difficulty. Without this pre-existing interest in gaming or Minecraft, youth may not show the same levels of motivation or engagement, and the programme is no longer ‘strengths-based’. As such, clinicians seeking to implement gaming-based programmes should consider the individual interests of each child or adolescent. At the same time, the intervention outcomes of the gaming-based programme and teaching strategies employed are essential for it to be neurodiversity-affirmative. For instance, gaming-based programmes can still be implemented with the explicit aim of targeting the reduction in autistic traits (Silva et al., 2021), thus while the gaming-based programme we report seems to be more neurodiversity-affirmative, essential elements, we describe, such as goal setting with participants, power-sharing, leveraging strengths and promoting authentic ways of communicating are essential.

Given the flexibility required in implementing these approaches, it is unlikely that a standardized protocol for implementing gaming-based programmes is possible, however, we believe that there are several recommendations that could be provided based on our context-mechanism-outcome framework. First, the implementation must be performed in a person- and family-centred manner. Therapists must engage with the child and their caregivers to determine interest and motivation prior to engaging in gaming-based programmes and to set individual goals. As caregiver involvement and perceptions of gaming can also influence implementation, therapists may benefit from providing education and resources to caregivers on the purposes and aims of the gaming-based programme, including how to support their child throughout the process. In implementing gaming-based programmes, therapists must remain cognizant of the aims of programme and their approach when designing the activities for the youth and providing support. Within gaming-based programmes, there may be risks that the support and intervention components become lost, and thus, therapists should be mindful of specific individual and group goals set by the youth and provide a safe space for these goals to be achieved. Our realist evaluation suggests that more organic and implicit support can facilitate outcomes such as independence and confidence. For example, facilitators should empower youth to problem-solve solutions together and form their own ways of effectively and authentically communicating rather than providing explicit instructions. Of course, within a person-centred approach, specific instruction or support may still however be required for specific incidents or tasks (for example in cases of distress or learning new skills such as compromise). Therapists should also be knowledgeable of the gaming platform to provide a foundation for shared interests with the youth, but should enable youth to sit within an ‘expert’ role. It should be noted that while these recommendations may provide some foundation for designing and delivering gaming-based interventions to autistic youth, these recommendations are based on a relatively small and homogeneous sample, thus these recommendations should also be empirically investigated using higher quality research designs.

### Limitations

This study has several limitations. The sample size is small and specific to only one service provider and one gaming-based programme, so it may not be representative of the experiences of all individuals engaging in gaming-based programmes. At the same time, although the gaming-based programme was available to neurodivergent youth regardless of gender or diagnosis, all youth were autistic and a majority were male. Thus, our sample represented a small and homogeneous group. On the other hand, the ethnographic approach, triangulation of data across multiple stakeholder types and extended and frequent observations contributed richness to the data. Due to our relatively small and homogeneous sample it is unknown if the contexts, mechanisms and outcomes identified within the current study translate to other neurodivergent youth, including other genders or individuals with other types of neurodivergence, as such findings and recommendations should be considered with caution, especially when extrapolating to other groups. Critically, it is important to note that social communication and interaction difficulties experienced by autistic individuals are not purely the result of social interaction difficulties within a person but are also the result of poor person-environment fits and the mismatch between autistic and non-autistic communication styles. For example, some evidence suggests that autistic individuals and non-autistic individuals are equally effective in communicating information within their respective neurotypes, suggesting that that is the cross-mixing of neurotypes that results in communication breakdown (Crompton et al., 2020). At the same time, autistic individuals face stigma, bullying and unfounded negative evaluations from neurotypical individuals (Sasson et al., 2017). For this reason, we do not propose that interventions, such as the current gaming-based programme are a panacea for all the difficulties faced by autistic youth where more widespread acceptance from non-autistic peers and environments is also needed. However, our findings suggest that such programmes may be helpful for some autistic youth, particularly where youth may benefit from practicing strategies to ensure that their needs and emotions are communicated to others. Gaming-based programmes may play a role in holistic approaches that target individuals (i.e. skills development, confidence) and broader contextual factors to support individuals.

## Conclusion

This study employed a realist evaluation to define the complex relationship between contexts, mechanisms and outcomes of a gaming-based programme for autistic youth. The results from this study highlight the significance of harnessing youth’s interest and employing a naturalistic setting to promote engagement in the intervention process and the development of social connection and competencies in a way that may be more neurodiversity-affirming and strength-based than other more traditional intervention and support programmes.

## Supplemental Material

sj-docx-1-aut-10.1177_13623613251320542 – Supplemental material for Gaming in the intervention and support process: A realist evaluation of a gaming-based programme Supplemental material, sj-docx-1-aut-10.1177_13623613251320542 for Gaming in the intervention and support process: A realist evaluation of a gaming-based programme by Melissa H Black, Abi Lilford, Vy Nguyen, Erin Walker, Haw HueiWee, Olov Falkmer and Sarah McGarry in Autism
