# Supplementary material for: Gaming in the intervention and support process: A realist evaluation of a gaming-based programme
Source: Autism. 2025 Mar 15;29(7):1688–99. doi: 10.1177/13623613251320542 (PMC12159337; doi:10.1177/13623613251320542)
Supplement: sj-docx-1-aut-10.1177_13623613251320542 – Supplemental material for Gaming in the intervention and support process: A realist evaluation of a gaming-based programme [file sj-docx-1-aut-10.1177_13623613251320542.docx]

| **Example quote** | **Category** |
| --- | --- |
| “And most of the clients, not all of them, but most of the clients that I work with, they are better at Minecraft than I am” | **Context** |
| “guess it's like a bit of a fun spin on the usual experience. And it's also quite a bit more, like socially dependent than in like, other just casual gameplay. So there's an element of that, that we're able to socialize and talk with each other. And yeah, kind of like coordinate with each other as well. Yeah, had a lot of fun with it.” | **Mechanism** |
| “He really battles with expressing how he’s feeling but now he will tell his friends “Actually, you’re upsetting me. So, I’m going to go instead of throwing my ipad across the room”... | **Outcome** |

**Supplementary table 1.** Example quotes for context, mechanism and outcome categories.
